# Influence of the Alcoholic/Ethanolic Extract of *Mangifera indica* Residues on the Green Synthesis of FeO Nanoparticles and Their Application for the Remediation of Agricultural Soils

**DOI:** 10.3390/molecules26247633

**Published:** 2021-12-16

**Authors:** Jhordi Bautista-Guzman, Rosa Gomez-Morales, David Asmat-Campos, Noemi Raquel Checca

**Affiliations:** 1 Facultad de Ingeniería, Carrera Ingeniería Ambiental, Universidad Privada del Norte, Trujillo 13011, Peru; jhordibautista@outlook.com (J.B.-G.); rosarush1999@gmail.com (R.G.-M.); 2 Dirección de Investigación e Innovación, Universidad Privada del Norte, Trujillo 13011, Peru; 3 Brazilian Center for Physics Research, R. Dr. Xavier Sigaud 150, Rio de Janeiro 22290-180, Brazil; nomifsc@gmail.com

**Keywords:** FeO nanoparticles, green synthesis, *Mangifera indica*, soil remediation, metal removal

## Abstract

The green synthesis of iron oxide nanoparticles (FeO NP) has been investigated using the extract in absolute ethanolic and alcoholic solvents 96% from the peel of the mango fruit (*Mangifera indica*), thus evaluating the influence of the type of solvent on the extraction of reducing metabolites. A broad approach to characterization initially controlled by UV-vis spectrophotometry has been directed, the formation mechanism was evaluated by Fourier transform infrared spectroscopy (FTIR), the magnetic properties by characterization by Physical Property Measurement System (PPSM), in addition to a large number of techniques such as X-ray energy dispersive spectroscopy (EDS), X-ray diffraction (DRX), transmission electron microscopy (TEM/STEM), electron energy loss spectroscopy (EELS), and Z potential to confirm the formation of FeO NP. The results suggest better characteristics for FeO NP synthesized using 96% alcoholic solvent extract. The successful synthesis was directly proven in the removal of metals (Cr-VI, Cd, and Pb) as a potential alternative in the remediation of agricultural soils.

## 1. Introduction

In recent years the use of nanoparticles (NP) has attracted the attention of the scientific community due to their profitability [1], efficiency [2], and a variety of physicochemical characteristics [3] as hollow colloidal nanoparticles that have pores for integral use of the surface and volume in order to carry out biological, physical, and chemical activities [4]. Likewise, they have a wide field of application such as in the textile industry [5], nanomedicine [6], soil remediation, water [7], and transformation and detoxification of a wide variety of environmental pollutants such as chlorinated organic solvents, polychlorinated biphenyls (PCBs), nitrates, bacteria, hydrocarbons, and heavy metals [8]. The synthesis methodology of iron oxide nanoparticles (FeO NP) can occur from different materials, but many of the adverse effects are associated with toxicity, due to the presence of absorbed substances on the surface of the nanoparticles [9,10,11]; in these cases, some work uses new mechanisms such as ‘nano zero valent’ iron nanoparticles (nZVI) that are stored in nitrogen gas to avoid oxidation during the synthesis or drying [12,13,14]. On the contrary, an oxygenic atmosphere favors the generation of magnetic NP Fe (Fe_3_O_4_ and Fe_2_O_3_) which stand out for their high specific surface and absorbent capacity, and due to their magnetism they enable the extraction of particles through external magnetic fields [15,16] and nanoparticles with superparamagnetic properties [17,18].

Thus, eco-friendly alternatives are methods using microorganisms, enzymes, fungi, and plant or fruit extracts. On the latter, there are investigations using *Parthenocissus quinquefolia* [19], green tea [20], coconut shell [21], eucalyptus [22], *Moringa oleifera* [23], and *Bauhinia tomentosa* [24]. The development of these ecosystem-friendly methods for nanoparticle synthesis has become an important branch of nanotechnology: “green synthesis” [25,26]. Plant or fruit extracts contain phytochemicals and metabolites that give them unique properties, which can be used as reducing agents for the synthesis and stabilization of nanoparticles [13,27].

According to [28], the synthesis of iron nanoparticles supported with bentonite obtained sizes between 40–80 nm, and the analysis of the nanomaterial shows the presence of C, O, and Fe, attributing the former to molecules and polyphenols of green tea extract. The absorption of Cr (VI) in sandy loam soil was stable up to a concentration of 100 mg/L; subsequently it decreased as a result of the availability of active sites for the adsorption of Cr, deducing an increase in the particle dose for higher concentrations of pollutant. The estimated pH to achieve good results is between 2–6 on the acid scale, and [29] obtained iron nanoparticles and carried out a comparative analysis of chemically synthesized nanoparticles with extracts of neem and mint leaves; the removal of lead and nickel at a concentration of 0.1 g NP/Kg of soil shows that the NP by NaBH_4_ removed 21.6% Pb and 18.5% Ni; while with neem extract 26.9% Pb and 33.2% Ni. The low removal efficiency is the product of agglomeration and exposure to the atmosphere resulting in oxidation of the surface of the particles. In the case of mint leaves, a concentration of 0.2 g NP/Kg of soil and the elimination of 66.1% Pb and 56.1% Ni was obtained. The improvement in performance is due to the little agglomeration between the nanomaterial, unlike the others, increasing the reaction area [30]. In the case [31] synthesized iron oxide nanoparticles with *Excoecaria cochinchinensis* extract which were applied to eliminate arsenic in soils, for the SEM-EDS characterization, nanoparticles between 50 and 100 nm with Fe compounds were evidenced, O, C product of the reduction mechanism. The BET surface area was 32 m^2^.g^−1^ and had a mean pore diameter of 12 nm, indicating high adsorption capacity. The adsorption of As occurred in the amorphous and crystalline Fe oxide layer, due to its positive charge that could adsorb both arsenate and arsenite simultaneously. FeO NP was obtained using *Excoecaria cochinchnensis Lour* extract with sizes between 5 and 20 nm with different carbon-oxygen functional groups on the surface of the Fe_3_O_4_ spheres, which contribute to the adsorption of Cd, and on the surface of the particles by electrostatic attraction. The adsorption rate was 93.69% at 30 min. The change in pH exerts variation in the removal of Cd (II): at low pH the elimination of Cd (II) decreases by increasing the temperature, indicating that the adsorption process is an exothermic process. Furthermore, an increased dose of nanoparticles and low temperature increase the adsorption rate [32]. Nanoparticles have a size between 38–47 nm and it was shown that they can oxidize As (III) to As (V) almost completely at 24 h, with pH values 3 and 7. On the contrary, [33] investigated the elimination of As (V) with nanoparticles synthesized with green tea extract which have a size between 50 and 100 nm, irregular and amorphous, and during the adsorption of As (V) the amount of active sites were reduced.

The objective of this research is to evaluate the influence of the solvent (96% alcohol and absolute ethanol) in the extraction of metabolites from the residues (shell) of *Mangifera indica* for its application as an organic reducer in the synthesis of FeO nanoparticles; likewise, the nanoparticulate material will be applied in the removal of metals present in agricultural soil such as hexavalent chromium (Cr-VI), cadmium (Cd), and lead (Pb).

## 2. Materials and Methods

### 2.1. Materials

Iron nitrate (Fe (NO_3_)_3_ 9H_2_O) (CAS: 7782-61-8, Merck, Germany) and absolute ethanol (CAS No. 64-17-5) were purchased from Merck Millipore, while alcohol 96% GL of the national chain of pharmacies ‘MiFarma’, in Peru. The samples of ripe mango (*M. indica* var. Edward) were acquired from a local supermarket in the city of Trujillo, Peru. Ultrapure water (Thermo Scientific, Barnstead Smart2Pure, Waltham, MA, USA) was used throughout the investigation.

### 2.2. Treatment of M. indica Husk

The *M. indica* fruits were washed with ultrapure water to remove impurities. Subsequently, the peel was peeled and separated. From a total of six mango units (2.08 kg), 307.7 g of wet peel was obtained, which was dried in a forced convection oven UNPA—MEMMERT model UM 55 plus (Memmert GmbH Co. KG. Schwabach, Germany) for 15 h at temperature of 75 °C to eliminate the humidity present. After the established time, we proceeded to crush, finally obtaining 34.11 g of powdered shell. The sample was stored at 4 °C in a refrigerator for use in subsequent procedures.

### 2.3. Obtaining Alcoholic Extract 96%/Absolute Ethanolic

With what was previously obtained, an aliquot of 5 g was taken of *M. indica* ground being diluted, in two beakers, with 50 mL of alcohol at 96% G.L. and 50 mL of absolute ethanol. Second, each sample was subjected to magnetic stirring (CAT-M6) in beakers at 300 RPM for 30 min. The mixtures were then placed in test tubes and subjected to an electric centrifuge (Hettich Zentrifugen, EBA 20C) at 3000 RPM for 15 min. Subsequently, the supernatants were filtered with a diaphragm vacuum pump (GAST DOA-P704-AA); finally, it was stored at 4 °C, covered with aluminum foil to avoid undesirable reactions and its subsequent use of FeO NP.

### 2.4. Green Synthesis of Iron Oxide Nanoparticles with Alcoholic 96% and Absolute Ethanolic Solvent Extracts

**Figure 1:**
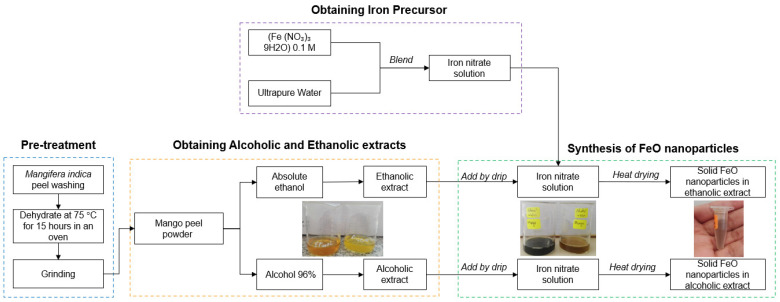
Block diagram of the green synthesis process of FeO NP mediated by *M. indica* extract.

For the synthesis in the green route (Figure 1), 100 mL of the precursor iron nitrate nonahydrate (Fe (NO_3_)9H_2_O) 0.1 M was prepared, which was distributed in volumes of 50 mL for each synthesis. Next, 6 mL of each extract was added dropwise in 96% alcoholic and absolute ethanolic solvents respectively, and it was kept under constant stirring of 400 RPM at room temperature. Subsequently, the solutions obtained were subjected to heat in an electric stove until reaching 120 °C. For this, the beaker with the mixture was placed in a larger baker glass with water up to half. Finally, the mixing beaker was removed when the liquid content was consumed in order to obtain solid nanoparticles.

### 2.5. Characterization of Iron Oxide Nanoparticles (FeO NP)

The absorbance spectra of FeO NP were obtained by UV-vis spectrophotometry (Hewlett Packard, 8452, Palo Alto, CA, USA) in the range of 300 to 900 nm, in order to find the typical wavelength of plasmon resonance surface (RPS) on iron oxide nanostructures, as well as to evaluate the stability of the nanoparticles over time. Additionally, the samples were analyzed using a Nicolet iS50 FT-IR infrared spectrometer (Thermo Fischer Scientific) to evaluate the organic agents present in the extracts and FeO NP and to respond to possible mechanisms involved in the formation of nanostructures. Electron microscopy analyses were performed using a Transmission Electron Microscopy (TEM) in the modes: High Resolution TEM (HRTEM), Scanning TEM (STEM), and Energy-Dispersive X-ray Spectroscopy (EDS) to characterize the morphology and elemental composition of the FeO nanoparticles. The studies were conducted in a JEOL 2100F equipment, operating at an accelerating voltage of 200 kV/current 130 µA and equipped with an CCD camera (One view). STEM images were obtained using an annular dark field (ADF) detector. The elemental composition was investigated by EDS using the STEM-DF mode and an Energy spectrometer of Oxford (Xplore).

The microscope was also equipped with electron energy loss spectroscopy (EELS) (EELS-GIF Tridiem GATAN) accessories. EELS spectra were conducted in the STEM imaging mode using a spot size of 0.7 nm. The spectrometer aperture was 5 mm. The energy resolution measured by the FWHM of the zero loss peaks was approximately 1.6 eV.

For TEM observation, the nanoparticles were placed inside a 1.5 mL tube with acetone and ultrasonicated for 30 min. The acetone solution was then dropped over carbon-covered TEM grids.

X-ray diffraction patterns of the nanoparticles were obtained in an Empyrean diffractometer (Panalytical) with Cu-Kα radiation (λ = 1.54056 Å) at 45 kV and 40 mA. Data were collected in the 20° < 2θ < 80° range in Bragg Brentano geometry, in spinner mode, with a step size of 0.026°.

The stability of FeO NP was characterized by Potential Zeta using the Zeta plus—Zeta potential analyzer equipment from the Brookhaven Instrument Corporation, from their electrophoretic mobility. These data are reported as a standard deviation and average of 10 different measurements.

Magnetic properties were measured using a Physical Property Measurement System (PPMS) DynaCool of Quantum Design at CBPF. The magnetization versus temperature measurements were performed in zero field cooled (ZFC) and field cooled (FC) conditions with a 200 Oe probe field. The hysteresis loops were measured at range 5 K and 300 K with an applied field up to 9 T.

### 2.6. Removal of Cr-VI, Cd and Pb Present in Agricultural Soil

Samples of 125 g of soil (from cultivation areas of the district of Moche, Trujillo in Peru), each diluted in 250 mL of ultrapure water, were worked. In order to evaluate the influence of the FeO NP volume on the removal of metals, a total of three samples C1 = 5 mL, C2 = 10 mL, and C3 = 15 mL were considered, the same ones that were volumetric with ultrapure water up to a 50 mL value. Subsequently, the samples were taken to the magnetic stirrer at 250 RPM for 30 min to obtain a correct homogenization between the FeO NP and the contaminated soil. Then, a 5 mL aliquot was taken from each of the samples, to which 10 mL of aqua regia (HNO_3_ + 3HCl) 1 M was added, and they were made up with deionized water until reaching a volume of 50 mL respectively. They were then subjected to the digestion method until the volume was reduced to 10 mL for approximately 50 min. Finally, the samples were volumetric with ultrapure water, up to the initial volume (50 mL), and were filtered with a diaphragm vacuum pump, until they were free of impurities. In addition, they were analyzed in the Atomic Absorption Spectrophotometer (Agilent Technologies, 200 series AA) for their reading.

## 3. Results and Discussion

### 3.1. UV-Vis Spectrum Analysis

Characterization by UV-vis spectrometry was considered as a preliminary analysis to show a synthesis of nanoparticles.

**Figure 2:**
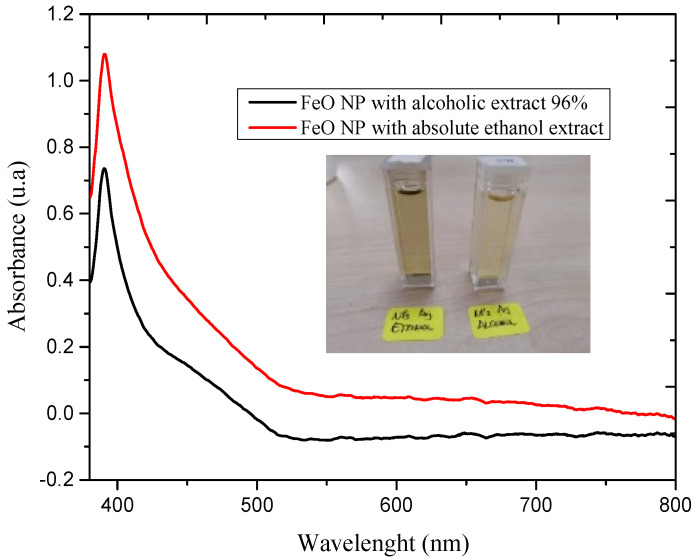
UV-vis spectrum of FeO NP obtained by the green route, using extracts of *M. indica* in different solvents. The inset shows the colloids FeO NP obtained.

The reduction of the precursor agent upon exposure to the extracts in the mentioned solvents is followed by characterization by UV-vis spectrophotometry, the spectra of which are shown in Figure 2.

The results indicate the presence of the RPS peak at 390.4 and 391.2 nm for FeO NP syntheses using 96% alcoholic and absolute ethanolic solvent extracts respectively, typical for this type of nanostructure. There is also evidence of a difference between the bandwidths and the absorbance, the FeO NP sample obtained with an extract in absolute ethanolic solvent being predominant, which shows a higher production of nanostructures, but at the same time a high polydispersity of sizes. These results certify the visual perception of the color change of the solution from light brown to black and describe the formation of FeO NP using *M. indica* extract, this being consistent with the UV-vis spectra of the nanoparticles under study.

Evaluating the stability over time is an important factor to identify the viability of the synthesis of nanostructures.

**Figure 3:**
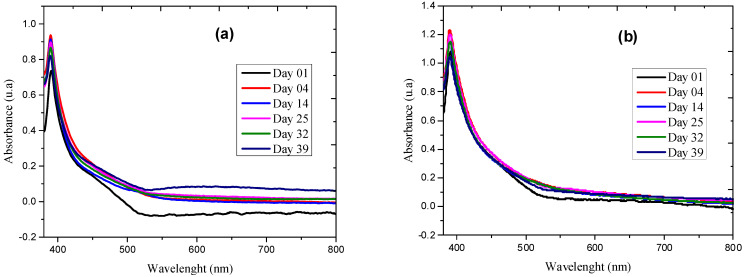
Evaluation of the stability of FeO NP by UV-vis spectrophotometry: (**a**) with extract in alcoholic extract 96% and (**b**) with extract in absolute ethanolic extract.

In Figure 3a,b, spectra are presented as a result of the sequential evaluation from day 1 of the synthesis to day 39 of the FeO NP. The results suggest a good behavior in the projection of the RPS peak on the axis wavelength, which leads us to consider that it is highly stable in both extracts, but with a slight improvement by the alcoholic solvent 96%. However, there is dynamic activity in the absorbances since the values increase compared to the first day, which means that there is a process of formation of more nanoparticles, possibly due to the fact that the green synthesis methodology does not usually have a total reduction efficiency of the precursor, which generates a greater production of nanostructures and therefore an increase in absorbance. For this case, Figure 3b shows a higher absorbance value.

Polyphenols are generally believed to be responsible for the synthesis and stabilization of iron oxide nanoparticles and are produced by complexing with the Fe salt and simultaneous reduction of Fe (III) with oxidized polyphenols [34].

### 3.2. Fourier Transform Infrared Spectroscopy—FTIR

Functional groups play a very important role in green synthesis. Therefore, to identify the involvement of these functional groups, the FTIR analysis was carried out before and after the synthesis of FeO NP using *M. indica* extract with the solvents under study.

**Figure 4:**
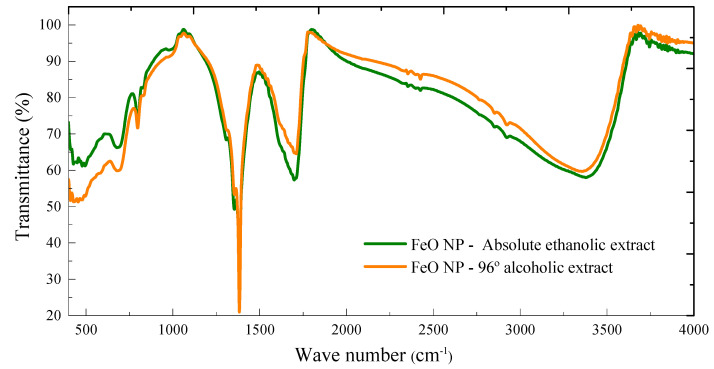
FTIR spectra of FeO NP using 96% alcohol solvent extract and absolute ethanol.

The FTIR spectra of the synthesized FeO NP using extracts in 96% alcoholic solvent and absolute ethanolic are shown in Figure 4. The samples show peaks in highly coincident ranges with values between 3371 and 3387 cm^−1^ which evidence the presence of OH stretching and aromatic and aliphatic CH respectively, 1697–1726 cm^−1^ corresponding to the stretching vibrations of the C=C and C=O bonds respectively. Likewise, there is a fairly noticeable peak in both samples at 1352 and 1382 cm^−1^, which is linked to the presence of calcium phosphates and CO and C=O groups, which can be evidenced in the characterization by EDX. The aforementioned peaks disappear when analyzing the FeO NP samples, which would allow us to approximate the mechanism of formation of nanostructures and specifically associated with the functional groups C=C and C=O that derive from water-soluble compounds such as flavonoids, terpenoids considered protective ligands of NPs [35,36]. Mango, being a fruit of tropical climate, has a quantity of these compounds including others such as flavonoids, gallates, benzophenones, and derivatives of gallic acid [37]. The peaks at 788 and 796 cm^−1^ are associated with the vibration of the FeO NP.

### 3.3. X-ray Diffraction—XRD

**Figure 5:**
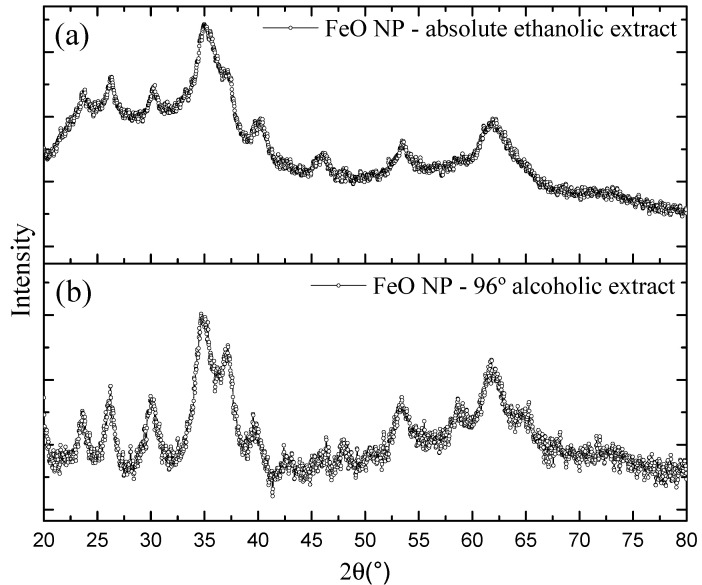
Characterization by XRD of FeO NP synthesized with extracts in solvents (**a**) ethanolic absolute, (**b**) alcoholic 96%.

The X-Ray Diffraction (XRD) method was used to investigate the material structure of the iron nanoparticles. Characterizations were obtained for both synthesis conditions, which are shown in Figure 5. The diffractogram shows in both cases strong peaks in the 2θ region at 35.6°, 54.2°, 63.1° that are linked to the planes (311), (422), (440). These peaks are indexed to the magnetite (Fe_3_O_4_) magnetic phases with a slight contribution from the maghemite phase (γFe_2_O_3_). Our results are analogous to previous studies that address the green synthesis of FeO NP [2,38,39].

The average crystallite sizes calculated from the FeO NP using the full width at half maximum intensity (FWHM) of the dominant peak in combination with the Debye-Scherrer equation were 3.95 and 2.93 nm, for the FeO NP cases synthesized with extract in absolute ethanolic solvent and alcoholic 96% respectively, which follow the same trend of the estimated sizes determined by STEM.

### 3.4. Elemental Composition

**Figure 6:**
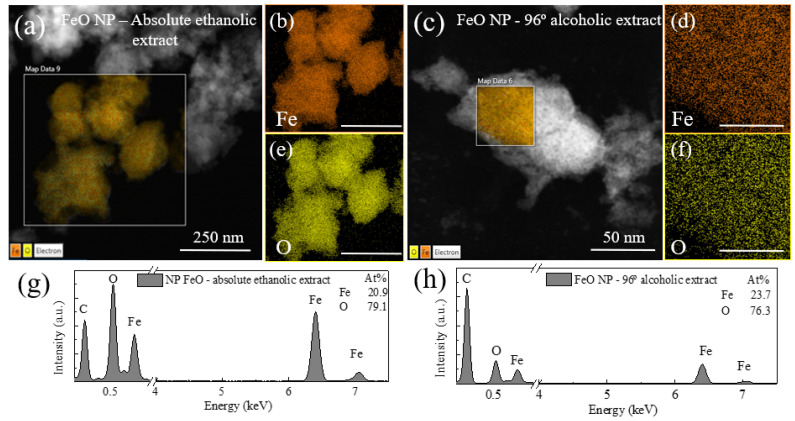
EDS spectrum of iron oxide nanoparticles synthesized using *M. indica* extract (**a,b,e,g**) absolute ethanolic solvent, (**c,d,f,h**) 96% alcoholic solvent.

The elemental analysis of the FeO NP by both methods was confirmed by the X-ray energy dispersive spectroscopy (EDS) analysis shown in Figure 6. The spectra show iron and oxygen peaks which indicate the presence of nanostructures of iron oxide with good relationship with XRD characterization. In the case of the sample using an extract in absolute ethanolic solvent, a higher content of iron (29%) is evidenced compared to the case of extract in alcoholic solvent 96% (23.7%), in both cases with a high oxygen content; in addition, at the first case, phosphorus and calcium were found, which was also evidenced in the FTIR characterization both in the extract and with a minimal reduction in the nanostructure. For the second case in question, no other elements were observed in the spectrum, which indicates the formation of pure NP Fe in oxide form, which was also verified in the results by FTIR where this peak disappears remarkably.

It should be mentioned that iron in its metallic form tends to react naturally with air (or water), which generates the formation of an iron oxide layer [40].

### 3.5. Transmission Electron Microscopy TEM/STEM

**Figure 7:**
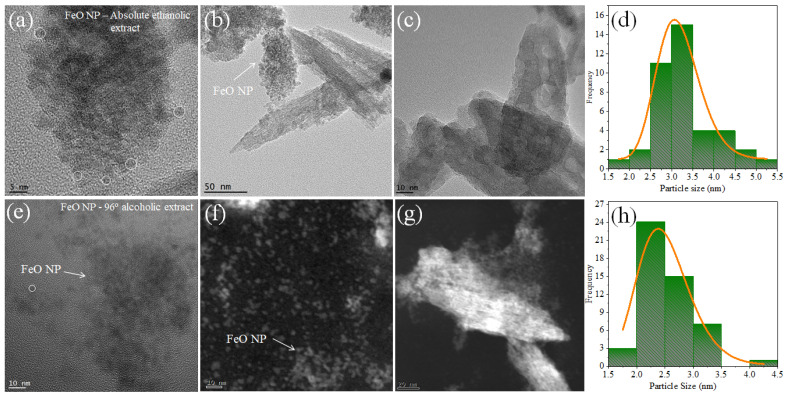
TEM/STEM images and size histogram of FeO NP synthesized using *M. indica* (**a**–**d**) with absolute ethanolic extract and (**e**–**h**) with 96% alcoholic extract.

The shape and size of the FeO nanoparticles synthesized by the green pathway were confirmed by TEM/STEM characterization. Figure 7a corresponds to the colloid obtained by using *M. indica* extract in absolute ethanolic solvent, and the image is shown in different magnifications, where the spherical morphology is evidenced and with an approximate size of 3.18 ± 0.51 nm. In the first image (5 nm scale) agglomerates are observed that are linked to organic materials present in the extract, this was also evidenced in the XRD diffractograms where there was a peak at 2θ at 26.2° attributable to organic stabilizing agents, in addition to the organic radicals shown in the FTIR.

The TEM image related to the FeO NP sample using 96% alcoholic solvent extract is presented in Figure 7b, where spherical morphology is also evidenced with an average size of 2.50 ± 0.47 nm.

The histogram shows the particle distribution and the mean size obtained. These dimensions coincide well with the results presented by UV-vis spectrophotometry, in addition to the results by XRD by calculating the size of the crystallite using the Debye-Scherrer equation. It can be seen that the size of FeO NP is affected by the type of solvent used for the extraction of metabolites from *M. indica*.

### 3.6. Characterization by Electron Energy Loss Spectroscopy (EELS)

**Figure 8:**
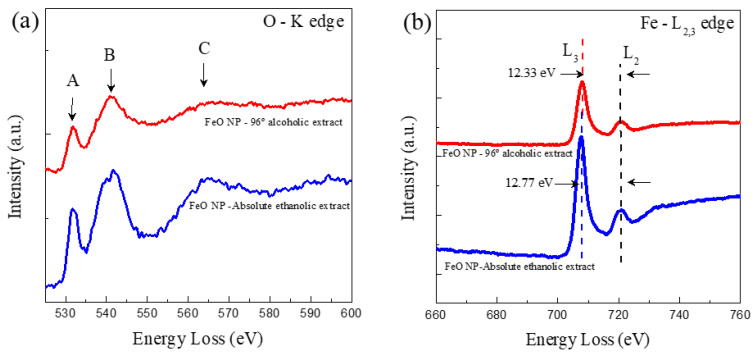
EELS spectra of O-K edges (**a**) and Fe-$L_{2,3}$ edges (**b**) acquired from a FeO NP 96% alcoholic extract (red curves) and FeO NP absolute ethanolic extract (blue curves), respectively.

The electronic structure of the FeO NP synthesized by the green route was analyzed. For this, extensive EELS experiments were carried out. Figure 8 shows typical EELS spectra for oxygen K edges and Fe-$L_{2,3}$ edges for FeO NP 96% alcoholic extract and absolute ethanolic extract. In both cases samples were collected from the central part. In Figure 8, three peaks are shown (labeled A–C), where the loss of energy near the edge of the fine structure can be identified in the K edge of oxygen; likewise, the intensities of the A peaks are shown and B for FeO NP synthesized with absolute ethanolic extract are lower than FeO NP with 96% alcohol. It has generally been shown that the intensities of the peaks are attributed to oxygen vacancies within the nanostructures; therefore, the lower the maximum intensity of the EELS spectra, the higher the content of oxygen vacancies in FeO NP. Given this, it can be deduced that the vacant oxygen content is higher in FeO NP synthesized with absolute ethanol extract of *M. indica* than using the extract with 96% alcohol.

Furthermore, the Fe-L edges provide us with the ionization state of the metal cations by identifying the individual edges $L_{3}$ y $L_{2}$, these results are shown in Figure 4. The general results indicate the presence of 78% Fe and 22% O for FeO NP using absolute ethanolic extract, while 52.03% Fe and 47.97% O for FeO NP using 96% alcohol extract. 

### 3.7. Characterization by Potential Zeta

The evaluation of the stability of the FeO NP was complemented by characterization by zeta potential from its electrophoretic mobility. For this, 1 mM KCl saline solution was used and adjusted to pH 6.5. The surface charge of the nanoparticles is considered an important characteristic since it is closely related to the stability of the colloids [41]. This is linked to the fact that it is a measure of the repulsion/electrostatic attraction between particles, in addition to being a predictor of stability.

For FeO NP with extract in ethanolic solvent, the average values calculated were 34.13 ± 3.03 mV and electrophoretic mobility 2.67 ± 0.24 (µ/S/V/cm), while for NP with extract in alcoholic solvent 96% the value was 22.01 ± 5.72 mV and electrophoretic mobility 1.72 ± 0.45 (µ/S/V/cm), which indicates high stability, and therefore lack of flocculation due to aggregation. This is also corroborated in the characterization by UV-vis spectrophotometry (Figure 3).

It should be noted that colloids with a high zeta potential (−/+) are electrically stable, whereas those with a low zeta potential (−/+) tend to coagulate [42]. Positive values could indicate that there is no excess presence of functional groups from the extract and some deprotonated biomolecules and therefore there is no coordination with the nanostructures obtained.

The zeta potentials obtained are comparable to other research works related to green synthesis [43,44].

### 3.8. Magnetization Measurements (SPION)

**Figure 9:**
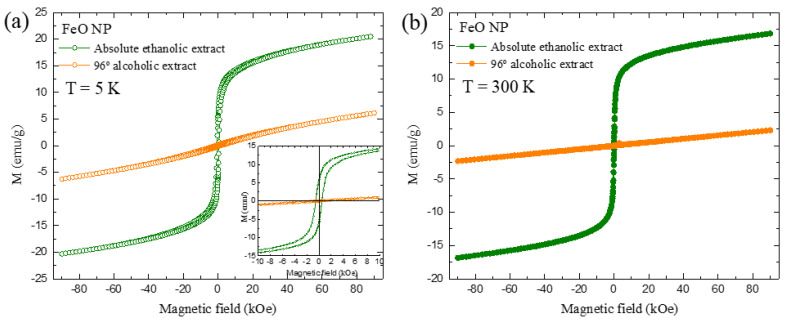
Measurements of saturation magnetization versus applied field hysteresis for FeO NP using extracts in ethanolic and alcoholic solvents (**a**) 5 K, (**b**) 300 K.

The magnetization curves are presented in Figure 9 (Ms vs. H). The results of both FeO NP samples exhibit a behavior attributed to superparamagnetic materials typical of SPIONS; in addition, the samples were evaluated at 5 and 300 K.

For 5 K, a maximum Ms was obtained around 20.5 emu/g and 6.13 emu/g. This decreased behavior was also evident when it was analyzed at 300 K, where values of 16.8 and 2.31 emu/g were obtained, all of the aforementioned for the extracts in ethanolic and alcoholic solvents respectively. In general, the magnetization values tend to decrease when there is also a decrease in the diameter of the nanoparticle. This behavior is corroborated in the results obtained by TEM/STEM and the size histogram with values of 3.18 and 2.50 nm for FeO NP using excerpts mentioned above.

The magnetic response values are lower compared to other methods such as thermal decomposition (76 emu/g) [45] and co-precipitation (60 emu/g) [46]. This reduction of Ms is linked to a crystalline disorder, spin inclination due to the reduction of the coordination of surface cations and/or negative surface effects promoted by a broken exchange between spins in NP with small crystallite size [2,47]. Regarding other green synthesis methods, Ms values lower than the one presented in this research have been reported (regardless of the type of solvent used to obtain the extract), such as 23 emu/g [48], 5.35 emu/g [17], 7.78 emu/g [49], 11 emu/g [18], 0.015 emu/g [50], and 1.57 emu/g [51].

**Figure 10:**
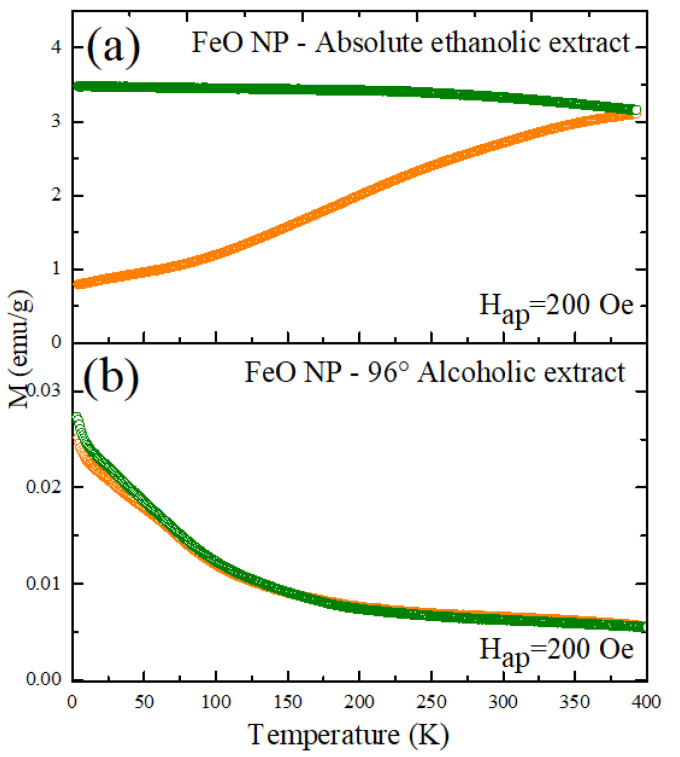
Magnetization curve as a function of temperature at low fields, under the ZFC and WFC protocols (**a**) FeO NP Absolute ethanolic extract, (**b**) FeO NP 96° alcoholic extract.

On the other hand, observed in Figure 10 is the curve of the magnetization as a function of the temperature of the FeO NP obtained by the green route with the two types of extracts mentioned. It can be seen that after cooling the sample under the influence of a magnetic field, both have a different response when varying the temperature. In the case of the sample with 96% alcoholic solvent extract, the increase in temperature generates a decrease in the magnetization values for both the zero field cool measurements ZFC (orange) and WFC (blue). This does not happen with the second sample, where it is evidenced that the ZFC behavior increases until it reaches its maximum point of 3.09 emu/g. The response of the WFC measure, by contrast, describes a decline. In both cases there is no evidence of thermal instability.

The green synthesis of nanoparticles is presented as a new and sustainable alternative to replace other typical synthesis methods where inorganic inputs are used throughout the process, which raises production costs and contributes to the contamination of both nature and the researchers who develop the methodology. However, in this type of ecological synthesis it is important to have a good management of the parameters linked to the process, in order to have nanostructures with similar or better characteristics than those developed by inorganic methods.

In this sense, this research provides as a first alternative the use of agro-industrial waste, specifically the peel of the mango fruit (*M. indica*), to extract the metabolites with the potential to reduce metal salts (precursor). Thus, the influence of the type of solvent used in the elaboration of the extract rich in bioactive compounds, such as absolute ethanol and 96% alcohol, has been evaluated. This is also linked to a decrease in production costs.

To consider a correct synthesis of nanoparticles, it is necessary to carry out a complete characterization that is evidenced from the analyses presented.

The results show a better result for the FeO NP synthesized using *M. indica* extract in alcoholic solvent 96%. This is how the spectrophotometry practiced for the evaluation of stability over time shows an invariance showing a slight increase in absorbance, possibly due to which an incomplete reaction was generated, which motivated us to continue with the reduction process and therefore resulted in a slight increase in the production of nanostructures, which later became stable with the passage of time. The FTIR characterization shows a possible mechanism involved in the reduction of the precursor agent (metal salt), with the C=C and C=O groups being the ones that lead to soluble compounds that act as reducing agents, such as flavonoids and terpenoids. It has been shown that the formation of this type of nanomaterial has led to having FeO phases such as magnetite and with a slight contribution of maghemite; likewise, the results of the elemental characterization (EDS) show that this type of sample has Fe and O without no other type of element. The morphology was evaluated by TEM/STEM, where the spherical type geometry is evidenced and with sizes close to the results obtained using the Debye-Scherrer equation from the XRD diffractograms. The result for zeta potential reinforces the fact of having achieved NP with high colloidal stability and the little participation of functional groups such as traces from the extract.

A behavior of decrease in the maximum values of magnetization between both methods has been evidenced. This is related to the variation of the diameters of the nanostructures, and because the magnetic properties are defined by characteristics such as size, surface, and the crystalline structure; in this sense, by obtaining a lower value of magnetic moment (emu/g) there is a possible alteration of the three-dimensional framework, motivated by structural defects and size that give rise to different magnetization values [52]. In general, the FeO NP obtained reach the saturation state governed by the superparamagnetic nature, which makes it very feasible to respond to magnetic fields without delay, indicating a material with high potential applicability in environmental remediation possess a perfect Langevin behavior that suggests the ability to act before an external magnetic field without maintaining a residual magnetism when the same field is eliminated, which further improves the various applications both in magnetic resonance imaging and cell separation [53,54,55].

On the contrary, the FeO NP obtained using *M. indica* extract in absolute ethanolic solvent show a high content of traces of organic material from the extract, since the characterization by FTIR and reinforced with EDS show the presence of phosphorus and calcium. It is also evident in the TEM/STEM images where there are very dense areas specifically linked to the aforementioned elements; likewise, UV-vis results show greater dynamics in the production of nanostructures with the passing of the days.

Thus, for the application in soil remediation, it has been considered to use FeO NP with pure content of this material without the implication of another type of element, in addition to considering smaller sizes and high monodispersity.

The agricultural soil used tests the influence of FeO NP in the removal of chromium (Cr-VI), cadmium (Cd) and lead (Pb). The results obtained are presented in Table 1. The removal of chromium (VI) with the 10 mL colloid (C2) obtained 99.48% removal during a period of 30 min. In this way, a reduction from 734.07 ppm to 3.815 ppm was achieved, which, compared to the Environmental Quality Standards (ECA) for agricultural soil in Peru [56], exceeds 0.4 ppm. Our research, like other studies, has obtained an efficient elimination of Cr (VI), and the formation of FeO nanoparticles and Fe (II) oxides is attributed to being strongly reducing. This is based on its various properties such as the modifiability of the surface by substances and functional groups, as well as its standard reduction potential, which for Fe2+/FeO is −0.44 V, being more negative than for Cr 6+/Cr3+(+1.51 V) [44,57,58,59]. In addition, the redox reactions of ferrous and ferric ions contribute to the process. The oxidation product binds pollutants through complex formation and ion exchange; iron oxides serve as a flocculant to capture metal ions and, therefore, remove contaminants present [60,61,62]. For the case of sample C1, there is no evidence of variation in metal concentration even after treatment; similar results indicate the decrease in removal due to the high initial concentration of Cr (VI) in the solution [63,64]. The effects between adsorbent dose and interaction time on chromium removal can prove lack of removal in the indicated sample. Additionally, they point to Cr (III) as the main responsible for forming a solid solution with iron, creating a passive layer of secondary phases such as magnetite, green oxide, goethite, and ferric hydroxide. This is attributed to the strong proton chemical gradient (Eh) that exists between the FeO surface and the surrounding medium. Minerals with Fe in a lower oxidation state, such as magnetite and green oxide, are often found close to FeO. The formation of such a passive layer decreases the rate of Cr (VI) reduction in the iron plate [65]. Therefore, increasing the initial concentration increases the degree of passivity and, therefore, decreases the reaction.

In the evaluation of the removal potential of cadmium, it presented a concentration of 0.2511 ppm, a value that complies with the ECA regulations for soil (1.4 ppm). The C3 sample (15 mL of colloid) reached the maximum removal of 81.48%, which translates to a concentration of 0.0465 ppm of cadmium in the analyzed test. Likewise, a proportional relationship between the amount of colloid and the percentage of removal achieved is evidenced, which means that as the milliliters of colloid in the sample decrease, the concentration in ppm increases. Studies affirm that the amount of adsorbent is a critical factor which determines the adsorption rate [66], thus there is a greater surface area of metal ions when the adsorbent dose is increased. Finally, in a similar way, the presence of lead was calculated and registered 497.27 ppm which decreased to 467.91 ppm, an approximate of 30 ppm between the initial and final concentration because the pH is an influential variable during the absorption of lead, it has been shown that pH < 4 protonation decreases the amount of active sites causing an electrostatic repulsion condition on the surface of the sorbent between H^+^ ions and Pb ions, causing less adsorption, likewise with pH > 6 it favors the generation of lead hydroxides which tend to precipitate [23,67]. A similar situation is evidenced with Cadmium according to the literature, where a deprotonation of the sample contributes to a better removal [68]. In our results, the high percentage of removal of the Cadmium C3 sample is also due to the low initial concentration for metal compared to Chromium and lead samples. However, the ideal pH situation for Chromium is contrary to the other two metals because it needs a high degree of H^+^ ions to achieve good removal efficiency. It has been shown that pH between 2 and 3 is usually the most suitable, because metal ions can replace H^+^ ions adsorbed on the surface of iron oxide nanoparticles through the ion exchange mechanism [69]. This can be explained by Equation (1) with the reduction of Cr (VI) to Cr (III) by Fe^2+^ action of Fe_3_O_4_ and subsequently coprecipitation in hydroxides (Equation (2)) [13,70].

(1) $${\mathrm{HCrO}}_{4}^{-}+3{\mathrm{Fe}}^{2+}+7H^{+}\to 2{\mathrm{Cr}}^{3+}+3{\mathrm{Fe}}^{3+}+4H_{2}O$$

(2) $$\left(1-x\right){\mathrm{Fe}}^{3+}+x{\mathrm{Cr}}^{3+}+2H_{2}O\to {\mathrm{Cr}}_{x}{\mathrm{Fe}}_{1-x}{\left(\mathrm{OH}\right)}_{3}+3H^{+}$$

In addition to the adsorption product of the electrostatic interaction of the pollutant and the magnetic nanoparticles, products of the availability on the surface of the nanomaterial, other removal mechanisms have been presented, such as chemical diffusion between adsorbent and adsorbate, surface precipitation, redox reactions, and ion exchange. In addition to removing inorganic contaminants, iron oxides can act as flocculants for the removal of colloidal organic substances in suspension [71]. We can mention the phytoremediation process, where a study tried to improve from the fusion of nanotechnology and photocatalytic degradation techniques to decontaminate soils. They obtained elimination of Pb, Ni, and Cd within 45 days after the phytoremediation study [72]. Compared to the method we used, the results were obtained in less time, which would be related to not considering plant species in the treatment.

## 4. Conclusions

It was determined that the influence of the use on the type of solvent for the extraction of reducing metabolites from the *M. indica* shell is linked to the degree of alcohol concentration, 96% being the one from which the most functional groups derived from soluble compounds such as flavonoids and terpenoids have been extracted, in addition to not affecting its properties. The FeO nanoparticles showed a spherical geometry with sizes between 2.5 and 3.1 nm for FeO NP in alcoholic 96% and absolute ethanolic solvents respectively. In addition, the XRD results confirm the presence of the magnetic phases of the magnetite as a slight contribution of the maghemite phase. The EDX results show that a maximum degree of alcohol concentration (absolute ethanol) generates the extraction of other elements (phosphorus and calcium) present in the extract and even in the nanoparticles. The magnetic response exhibits in both cases a behavior attributed to superparamagnetic materials, indicating the ability to respond to magnetic fields without delay, being a factor involved in the removal of metals. The successful synthesis of nanoparticles was applied in the removal of metals, achieving maximum values of 99.48% for chromium, 81.48% cadmium, and 5.9% lead.

## Figures and Tables

**Table 1:** Atomic absorption results in agricultural soil samples for chromium, cadmium, and lead; influence in function of FeO NP.

	CHROMIUM				CADMIUM				LEAD			
Sample	Abs.	Initial ppm	Final ppm	Removal (%)	Abs.	Initial ppm	Final ppm	Removal (%)	Abs.	Initial ppm	Final ppm	Removal (%)
**C3**	0.0046	734.07	3.87	99.47	0.008	0.2511	0.0465	81.48	0.16	497.27	467.91	5.90
**C2**	0.0045		3.815	99.48	0.0010		0.1488	40.74	0.18		526.63	0
**C1**	1.17		734.07	0	0.013		0.3023	0	0.18		526.63	0

## Data Availability

Not applicable.

## Sample Availability

Samples of the compounds are not available from the authors.
